# Methylation pseudotime analysis for label-free profiling of the temporal chromatin landscape with long-read sequencing

**DOI:** 10.1101/2025.03.03.641287

**Published:** 2025-03-11

**Authors:** Annie Trinh, Navied Akhtar, Kwadwo Bonsu, Nandor Laszik, Asia Mendelevich, Tanye Wen, Julien L. P. Morival, Katelyn E. Diune, Mitchell Frazeur, Justin E. Vega, Alexander A. Gimelbrant, Elizabeth L. Read, Timothy L. Downing

## Abstract

Faithful epigenetic inheritance across cell divisions is essential to maintaining cell identity and involves numerous epigenetic modifications, whose roles in establishing chromatin architecture are less understood. Technological approaches to temporally order epigenetic modifications throughout the cell cycle often face limitations in sequence resolution and rely on potentially damaging mitotic labeling or conversion steps. Herein, we present M ethylation P seudotime A nalysis T hrough read-level H eterogeneity (MPATH), a label- and conversion-free method to infer post-replication DNA strand maturity from methylation patterns across single molecules. We use MPATH to temporally order hydroxymethylation throughout mitotic inheritance, revealing that CpGs within cis-regulatory elements undergo transitions between methylation states at sub-cell-cycle timescales. When applied to long reads generated by NOMe-seq, MPATH uncovered relationships between nucleosome occupancy and DNA maturity. Finally, extension of MPATH to phased reads reveals allele-specific trends in pseudotime distribution associated with X chromosome activity. Our findings suggest that when coupled with multimodal sequencing strategies, MPATH could provide valuable insights into chromatin restoration dynamics.

## Full Text Availability

The license terms selected by the author(s) for this preprint version do not permit archiving in PMC. The full text is available from the preprint server.

